# Frequency and timing of renal biopsy complications 

**DOI:** 10.22088/cjim.15.1.10

**Published:** 2024

**Authors:** Maryam Pakfetrat, Leila Malekmakan, Anahid Hamidianjahromi, Razieh Rastegar

**Affiliations:** 1 Shiraz Nephro-Urology Research Center, Shiraz University of Medical Sciences, Shiraz, Iran

**Keywords:** Hematuria, Major complication, Minor complication, Percutaneous renal biopsy

## Abstract

**Background:**

Percutaneous renal biopsy is the primary diagnostic tool for renal diseases. In this study, we evaluated renal biopsy complications and the timing of complications.

**Methods:**

A cross-sectional study was performed on adult patients who underwent renal biopsy. The data gathering sheet collected patient characteristics. Complications were categorized as minor and major which needed an intervention. Data were analyzed using SPSS, and a p-value <0.05 was considered significant.

**Results:**

This cross-sectional study was conducted on 215 patients (mean age: 33.1±16.4 and 54.4%: women) who underwent percutaneous renal biopsy in Shiraz Nemazi Hospital for one year. Of the 298 complications that occurred, 90.2% were minors (56.1%of them microscopic hematuria). Moreover, 2 (0.7%) patients developed major complications and received a transfusion. In addition, most of the complications (98.9%, 295 ones) developed within 8 hours post-procedure. Only hemoglobin drop was significantly higher in women (41.0% vs. 21.4%, P=0.003).

**Conclusion:**

This study indicates that renal biopsy is a safe procedure; the results revealed that the significant post-biopsy complications were rare and occurred in the first 8 hours.

Percutaneous renal biopsy is the critical principle tool in diagnosing and treating renal diseases, which was first done in the 1950s by Iversen and Brun in Denmark (1). In 1954, it was modified by Kark and Muchercke to the prone position, which was more satisfactory (2). The improvement in biopsy techniques and equipment have made it a safe procedure with common life-threatening complications over time (3). 

Several studies have been conducted to evaluate the incidence of post-biopsy complications based on time, which helps determine the appropriate post-procedure monitoring duration (4, 5). In accordance with the 2019 guideline, it is recommended to observe patients for 6 to 24 hours after biopsy for early diagnosis of any complications (6). Post-renal biopsy complications are classified as either major or minor. Significant complications are defined as those that require intervention for resolution, and minor complications are the ones that spontaneously resolve (7).

Although our center is the referral in the south of Iran, where renal biopsies perform, no studies have been conducted so far. In this study, we decided to evaluate the frequency and timing of renal biopsy complications done in Shiraz Namazi Hospital for one year. 

## Methods

**Study design and population: **A cross-sectional study was performed on hospitalized adult patients who underwent percutaneous renal biopsy in Shiraz Nemazi Hospital, Shiraz University of Medical Sciences, Iran. 

**Study Protocol:** We prepared a data gathering sheet for patients, including; gender, age, the reason for biopsy, blood pressure, and basic laboratory tests like hemoglobin (Hb), platelet count, prothrombin time (PT), activated partial thromboplastin time (PTT), serum creatinine. All biopsies were done by nephrology fellows with 18-gauge needles using ultrasound-marked blind or real-time ultrasound-guided techniques. The preferred position for biopsies was prone (in native kidney patients) or supine (in transplant kidney patients). After the procedure, patients were instructed to maintain a supine posture in bed for 4 to 6 hours and recommended bed rest under close observation for 24 hours. Vital signs were checked every 15 minutes in the first hour, every hour for 4 hours, and then every 4 hours after that. All post-biopsy urine was checked for macroscopic hematuria, and after 6 hours, a urinalysis test was performed. Hb checked every 6 hours till 24 hours post-biopsy. Post-biopsy sonography was performed in all patients. If there was a hematoma in sonography, the patient was followed by Hb, urinalysis, and clinic. CT was performed as needed, including massive hematoma and unpredictable clinical or paraclinical. Complications were categorized as minor and major. Minor complications were those that did not require intervention, and major ones included those which needed any intervention, including surgery, angiography, or blood transfusion. The data of this study were collected in the first 24 hours after the biopsy; however, patients were examined clinically, and if they had any symptoms, such as fever, bleeding, or hematoma, they were not discharged.

**Definition:** The decrease in Hb determines Hb drop≥ 1g/dl. Hypotension is characterized by a symptomatic reduction in systolic blood pressure (< 90 mm Hg) (8, 9).

**Ethical issue:** The study was done in accordance with the Declaration of Helsinki and approved by the local ethics committee of Shiraz University of Medical Sciences. Informed consent was obtained verbally.

**Statistical analysis:** Statistical Package analyzed data for the Social Sciences software Version 23.0 (SPSS Inc. Chicago, IL). Qualitative data are expressed as numbers and percentages, which are analyzed by the chi-square. Quantitative data were presented as mean and standard deviation and analyzed by independent t-test and ANOVA Test. A p-value of less than 0.05 was considered statistically significant. 

## Results

Two hundred fifteen patients underwent percutaneous renal biopsy during the one year in Shiraz Nemazi Hospital. The mean age of patients was 33.1±16.4, of which 54.4% of them were women. Primary coagulative serum studies measured before biopsies (Hb, PLT count, PT, PT) were normal (table1). The two most common indications for renal biopsies were acute kidney injury (99 cases, 46.0%) and nephrotic-range proteinuria (78 patients, 36.3%). Thirty-two biopsies (14.8%) were done in patients with transplant kidney (table 2). 

**Table 1:** The studied patient's baseline data before renal biopsy

**Pre-biopsy characteristics**	**Measures**
**Hemoglobin **(g/dL), mean±SD	11.3±2.1
**Creatinine **(mg/dL), mean±SD	1.9±2.2
**Platelet count **(×10^3^/mL), median	130000.0
**PT**(second), median	13.0
**PTT**(second), median	33.0
**Systolic blood pressure **(mmHg),mean±SD	135.0±17.4
**Reason of biopsy**, n (%) **Acute kidney injury** **Nephrotic syndrome** **Both of them**	99 (46.0) 78 (36.3) 38 (17.7)

n: Number, Mean ± SD: Mean and Standard deviation, PT: Prothrombin time, PTT: Partial Thromboplastin Time.

**Table 2:** The studied patient's characteristics regarding complications after renal biopsy

**Characteristics**	**Total, **N=215	**Complication** Yes (n=196) No (n=19) p-value		
**Age**, year, (mean ± SD)	33.1±16.4	33.3±16.5	31.4±16.1	p>0.05
**Gender**, Women, n (%)	117 (54.4)	106 (59.1)	11 (57.8)	p>0.05
**Transplanted kidney**, n (%)	32 (14.8)	27 (13.7)	5 (26.3)	p>0.05

A total of 196 patients (91.2%) developed a complication. A total of 298 complications occurred among our patients, of which 295 (98.9%) developed the complication within 8 hours post procedure (table 3). The rate of minor complications was about 90.2% which were microscopic hematuria (56.1%) and macroscopic hematuria (42.8%), the two most common minors. And also, severe flank pain (2.0%) was the least common minor complication. In addition, 2 (0.7%) patients received transfusion due to Hb drop, categorized as a significant complication. The results showed no significant correlations between the rate of complications and demographic variables, except for the Hb drop that was significantly higher in women (41.0%) than in men (21.4%) (P=0.003). 

The timing of complications is shown in table 3. 98.9% of complications happened within 8 hours post-procedure, and only 0.7% (2 cases) were major ones. Approximately 1.0% (3 patients) complications occurred after 8 hours post-biopsy.

**Table 3:** The distribution of existing complications after renal biopsy

**Complication**, n (%)	**Total**	**Time of post biopsy presentation**		
		≤8 hours 9-24 hours >24hors		
**Minor complication** Microscopic Macroscopic Hemoglobin drop Sub capsular hematoma Hypotension Severe flank pain	296 (99.3) 110 (37.1) 84 (28.3) 69 (23.3) 15 (5.1) 12 (4.0) 6 (2.0)	293 (98.3) 110 (37.1) 84 (28.3) 69 (23.3) 15 (5.1) 10 (3.4) 5 (1.7)	2 (0.7) - - - - 2 (0.7) -	1 (0.3) - - - - - 1 (0.3)
**Major complication** Blood transfusion	2 (0.7) 2 (100.0)	2 (0.7) 2 (100.0)	- -	- -
**Total**	298 (100.0)	295 (98.9)	2 (0.7)	1 (0.3)

## Discussion

In this cross-sectional study of 215 patients, we evaluated the incidence of PRB complications in a referral center. Although percutaneous renal biopsy (PRB) can be associated with severe life-threatening complications, none of our cases required emergent surgery, angiography, or nephrectomy occurred. Most of the complications happened within 8 hours post-biopsy.

**Rate of complications:** Among our patients (all had pre-biopsy normal laboratory data), 298 PRB complications occurred, which only 2 of them were major ones that were treated with blood transfusions, and 90.2% were minor complications. The most common complication was microscopic hematuria (56.1%). Manno et al. performed a cohort study of 471 renal biopsies in 2004, of which 34.1% became complicated (7). The incidence rates of minor and major bleeding complications were 32.9% and 1.2%, respectively. In 2012, Tondela, et al. reported that 2.7% of patients experienced any complications, which was major in 1.0% of them (10). In a five-year study by Tuladhar, et al. in 2014, on 75 PRB patients, no major complications were reported and the rate of minor complications was 4.0% (11). In 2016, Parrey, et al. conducted a prospective study on 345 PRB patients during four-year period (12). 4.3% of patients experienced major complications and they received blood transfusion. In 2017, Yaqub et al. performed a ten-year period study on total 433 PRB patients with post-biopsy complication rate of 14.2% of which 4.8% was due to major ones (13). In 2017, Azmat, et al. studied on 220 PRB patients during 1-year period and reported the total post-biopsy complications as 19.1% (7.4% major and 11.7% minor), (14). The most common reported complication was gross hematuria (52.3%). In 2017, Roccatello, et al. in a prospective observational study reported the rate of PRB complications of total 462 biopsies during five-year period as 7.8%, which were 1.9% major and 5.8% minor (15). In 2019, Pombas, et al. (16) performed a study on 661 PRB patients of which 16.6% were associated with any complications (15.1% were minor and 1.5% were major), (16). In 2019, Trajceska L, et al. (17) analyzed 345 biopsies in 3 consecutive years, which 6% patients developed a complication (4.4% minor and 1.7% major), (17).

The reasons for this difference may be related to the needle gauge size, as 18-gauge needles are associated with more complications (10), the number of passes, and the technique we used (blind biopsies are associated with more complications than real-time ultrasound-guided biopsies (15). 

While some other studies reported the most common difficulty was gross hematuria, like ours (14), others presented that perinephric hematoma and pain were their most common complications (12, 16). 

A study also showed that complications were significantly higher in those with pre-biopsy prolonged aPTT, hypertension, body weight, low Hb, and nephrotic-range proteinuria (16, 17). In contrast, our cases had pre-biopsy normal laboratory studies.

**Timing of complications:** In our experience, most complications took place within 8 hours post-biopsy. Only three minor complications were identified after 8 hours, in which 2 cases of hypotension and 1 case of severe flank pain. Our results confirm the findings of the Carrington et al.’s study of 192 low-risk PRB patients in which all complications occurred 8 hours post-biopsy (18). Furthermore, in Schorr et al.’s study, the results showed that most complications (92.4%) happened immediately after biopsy and no major complication occurred after 5 hours of the procedure (19). Three other studies conducted by McMahon et al. with 105 cases (20), Al-Hweish, et al. with 44 cases^21^^,^ and Lin et al.^22^ also confirmed that most of the PRB complications in low risk patients happen within 5-6 hours post-biopsy (21, 22). Some other studies recommended post-biopsy observation of at least 12 hours to identify 80-90% of complications (3-4, 23). Compared to previous studies, patients are at higher risk of bleeding due to abnormal coagulation studies, lower Hb levels, uncontrolled blood pressures, and/or worse renal functions. In Jones B et al’s. study, 66% of complications were apparent within 6 hours of observation, while no complications occurred after 12 hours of the procedure (24). 

This study's lack of information was due to the retrospective nature of the research and no registry system for this issue in our center. 

Current results showed that the major PRB complications in low-risk patients are uncommon and often identified within 6 to 8 hours post-procedure. While chronic kidney disease has been recognized as one of the most current risk factors for severe COVID-19 disease and its associated mortality, unnecessarily prolonged hospitalization of these patients will undoubtedly increase exposure and transmission of the infection. Our recommendation is to categorize patients based on their risk factors to determine the optimal hospital observation period 6-8 hours for low-risk patients vs. 12-24 hours for high-risk ones) for each patient to minimize the exposure and transmission of infection. 
